# Ecological Characteristics and Nutritional Values of Australia-Native Brown Algae Species

**DOI:** 10.3390/md23100383

**Published:** 2025-09-26

**Authors:** Chao Dong, Cundong Xie, Ziqi Lou, Zu Jia Lee, Colin J. Barrow, Hafiz A. R. Suleria

**Affiliations:** 1 School of Agriculture, Food and Ecosystem Sciences, Faculty of Science, The University of Melbourne, Parkville, VIC 3010, Australia; 2 Centre for Chemistry and Biotechnology, School of Life and Environmental Sciences, Deakin University, Waurn Ponds, VIC 3125, Australia

**Keywords:** polyunsaturated fatty acids, polysaccharides, trace elements, brown seaweed, bioactivity

## Abstract

This review focuses on five native Australian brown algae species—*Cystophora torulosa*, *Durvillaea potatorum*, *Ecklonia radiata*, *Hormosira banksii*, and *Phyllospora comosa*—evaluating their environmental adaptability, biochemical composition, bioactive compounds, and potential for commercial development. Species-specific differences in temperature and light tolerance influence their habitat distribution. Nutritional assessments reveal that these algae are rich in proteins, polysaccharides, polyunsaturated fatty acids, and essential trace elements. Bioactive compounds, including polyphenols and fucoidans, exhibit antioxidant, anti-inflammatory, and anti-diabetic properties. *D. potatorum* extracts have considerable economic value in agriculture by enhancing crop yield, improving nutritional value, and promoting root development. *C. torulosa* is predominantly found in cooler marine environments and is comparatively more thermally sensitive. In contrast, *H. banksii* has a higher heat tolerance of up to 40 °C and thrives in warmer environments. *E. radiata* is widely distributed, highly tolerant of environmental stresses, and exhibits notable disease-resistant activities. *P. comosa*, due to its high polysaccharide content, demonstrates strong potential for industrial applications. Consumer studies indicate growing acceptance of seaweed-based products in Australia, although knowledge gaps remain. This study highlights the need for continued research, optimized processing methods, and targeted education to support the sustainable development and utilization of Australia’s native brown algae resources.

## 1. Introduction

Macroalgae, commonly referred to as seaweeds, are large multicellular marine algae that are photosynthetic aquatic eukaryotes [1]. They represent more than 90% of marine plant species, play a vital role in global oxygen production, and are responsible for over half of marine photosynthesis. As such, macroalgae are considered one of the most crucial marine resources for human survival [2]. Brown seaweeds are the most predominant type of macroalgae and represent the only group of heterokont protists that exhibit complex multicellularity [3]. Seaweeds are rich in proteins, carbohydrates, minerals, phenolic compounds, and various bioactive substances, including vitamins A, B, C, D, and E [4]. Due to their high nutritional value and potential in preventing chronic diseases such as cardiovascular disease and hypertension, seaweeds are widely utilized in food processing, cosmetics, pharmaceuticals, and other industrial applications [5,6].

As a food source, seaweed has been a significant component of human dietary traditions, particularly in Asia [7]. Archaeological evidence indicates seaweed consumption in Japan as early as 6000 BC (jōmon period) [8], where remnants of seaweed and fish bones have been discovered in ancient settlements. In China, seaweed became part of the regular diet by 2700 BC and was even offered as a royal tribute [9,10]. Beyond Asia, countries and regions such as Iceland [11], Ireland, Wales, Austria, Spain [12], and parts of the Americas [13] also have longstanding traditions of seaweed consumption [14]. France ranks as one of the most significant seaweed-producing and -consuming nations in Europe [15,16]. Indigenous communities in New Zealand [17] and Australia [18] have historically incorporated seaweeds into their diets as well.

The global seaweed industry has shown remarkable growth and holds significant potential for future development. Seaweed cultivation contributes to environmental sustainability and plays a strategic role in addressing global food security concerns. The algae industry is currently valued at over USD 15 billion and continues to grow steadily [19]. According to the United Nations World Aquaculture Statistics [19], seaweed farming accounted for 30% of global aquaculture production in 2020, with a total output of 35.1 million tonnes. Nearly 100 countries are involved in seaweed cultivation, with Asia contributing 98.9% of global production, demonstrating both the economic viability and ecological adaptability of seaweed as a food resource.

Australia, with nearly 20,000 km of coastline [20] and more than 71,000 square kilometers of underwater algal forests [21], is home to over 1200 species of macroalgae [22,23]. These species exhibit high levels of diversity and endemism, positioning Australia as a prime region for seaweed cultivation. Despite this potential, Australia imports over AUD 40 million worth of seaweed annually [24], indicating underutilization of its native resources. To unlock the potential of Australia’s seaweed industry, further research is needed to explore the ecological and economic potential of local species. Enhancing domestic cultivation and processing capabilities could support the development of a sustainable seaweed sector and increase the value of related industries.

Seaweeds belong to three groups, red, green, and brown algae, based on morphology, pigment composition and chemical characteristics. Brown algae comprise around 2000 species and contribute to over 66% of total seaweed consumption worldwide [25,26,27]. This review focuses on five Australian brown algae species—*Cystophora torulosa*, *Durvillaea potatorum*, *Ecklonia radiata*, *Hormosira banksii*, and *Phyllospora comosa*—and discusses their ecological characteristics, nutritional value, and the current landscape of seaweed consumption and cultivation in Australia.

## 2. Ecological Characteristics of Five Australian Native Brown Algae

Five endemic brown algae species native to Australia were selected to evaluate their potential commercial value as sustainable economic resources for the food and nutrition industries. These species—*Cystophora torulosa*, *Durvillaea potatorum*, *Ecklonia radiata*, *Hormosira banksii*, and *Phyllospora comosa*—were chosen for their ecological significance, edibility, and palatability [28]. They exhibit characteristics favorable for sustainable harvesting, support the stability of local marine ecosystems, and hold promise as future contributors to the brown algae aquaculture industry in Australia. The morphological characteristics and geographical distributions of these species are summarized in Table 1.

### 2.1. Cystophora torulosa

The genus *Cystophora* comprises 26 known species, all endemic to Australian waters, and forms a major component of the subtidal macroalgal forests in southern Australia [29]. *Cystophora* species are primarily distributed in cooler marine environments, typically with average sea temperatures below 20 °C. While the overall distribution pattern has remained relatively stable, some species have declined or disappeared due to climate-related changes [30]. Intensive regional sampling revealed a sharp decline in *Cystophora* species populations in warmer zones since the 1980s. For example, no species were detected in Perth, and only limited individuals of *C. brownii* were observed in Jurien Bay and Lancelin [30,31,32]. Heatwave surveys in the 21st century further demonstrated that *Cystophora* species were only present in areas where the mean temperature remained below 20 °C, with no reappearance in coral reef habitats following thermal disturbances. Certain species even experienced population declines of up to 8%. Growth in this genus is negatively correlated with light intensity and temperature, highlighting its high thermal sensitivity [30,33,34,35].

*Cystophora torulosa* is a perennial monoecious brown algae distributed mainly across the intertidal and subtidal zones of southeastern Australia. It prefers sheltered environments and exhibits poor tolerance to dry or sun-exposed conditions. Its reproductive period typically occurs from September to December [29,36]. A 12-month assessment of temporal and spatial variation in *C. torulosa* revealed no significant differences in distribution, abundance, or reproductive activity. Thallus length reached its maximum in June (winter), while levels of biofouling remained negligible [37]. These findings suggest that *C. torulosa* may represent a reliable candidate for future commercialization.

### 2.2. Durvillaea potatorum

The genus *Durvillaea* includes at least six known species of large, dioecious brown macroalgae that reproduce through gamete fusion [38]. These species are confined to the Southern Hemisphere and occur primarily in low intertidal zones of Australia, New Zealand, Chile, and adjacent regions. *Durvillaea* species typically attach to rocky substrates in wave-exposed environments and play critical roles in maintaining local biodiversity and ecosystem stability [38,39,40,41]. These species are vulnerable to prolonged exposure to sunlight and ice scour events [41]. Thomsen et al. [42] reported that during the 2017/2018 marine heatwave, prolonged sunlight exposure (>3 h) at sea surface temperatures above 23 °C (with atmospheric temperatures exceeding 30 °C) led to the local disappearance of *Durvillaea* species. This loss was followed by the invasion of non-native algae (*Undaria pinnatifida*), potentially threatening native ecological balances [42,43].

*Durvillaea potatorum* is a commercially valuable Australian species widely distributed around King Island, Tasmania, and western Wilsons Promontory [44]. Specifically, *Durvillaea potatorum* is widely distributed along the eastern, western, and southern coasts of Tasmania, and in Victoria, its range extends from the northeastern boundary to Robe in the west [38]. Similar to other *Durvillaea* species, its growth relies heavily on firm attachment to rocky substrates, and it thrives in wave-exposed environments due to its non-buoyant morphology [38,45]. It tolerates strong wave action, is primarily harvested from King Island under regulated conditions, and has been observed to make up a greater proportion of samples collected from intertidal areas [38]. In Tasmania, commercial harvesting is prohibited to preserve local ecosystems and fisheries [46].

Distinctive morphological features that differentiate *Durvillaea potatorum* from other species include the absence of stipitate lateral blades from the lamina margins or primary stipe, inflated mature laminae with honeycombed tissue, and internally excavated holdfasts [47]. In addition to its ecological significance, *D. potatorum* is believed to be the bull kelp traditionally used by Aboriginal communities in Tasmania, valued for its intact and rarely branching palmate fronds, which were woven into water carriers and baskets [48]. Beyond these traditional applications, recent studies have underscored the potential of *D. potatorum* in modern agriculture. Extracts of *D. potatorum*, when combined with *Ascophyllum nodosum*, have been shown to promote root growth by 22% and increase crop yields by 8–19%, offering a promising alternative or supplement to conventional soil fumigants, which are associated with adverse environmental effects [49]. More recent studies corroborate these findings, demonstrating that such extracts not only enhanced yields by 8–10% but also reduced the incidence and severity of post-harvest rots in strawberries by 52–87% [50].

### 2.3. Ecklonia radiata

The evolutionary history of *Ecklonia radiata* is relatively recent, particularly along Australia’s east coast [51,52]. Previously, species within the genus were classified into separate families due to limited phylogenetic data, but recent studies have clarified relationships within the genus. Notably, *Ecklonia* species from the northern and southern hemispheres exhibit distinct evolutionary lineages. The southern species, excluding *E. maxima* from South Africa, are now classified as *Ecklonia radiata* [53].

*E. radiata* is widely distributed throughout Australia—from the western to the eastern coast—and is known for its high environmental tolerance and adaptability to diverse physical conditions [54,55,56]. It is a relatively small dioecious kelp, usually less than 2 m in length, found in the temperate waters of South Africa, Australia, and New Zealand at depths of up to 40 m [56,57]. Morphological variation is observed across different geographic regions. It is considered a warm-temperate species, exhibiting optimal growth and reproduction at 12–20 °C, with performance declining outside this range [58]. For instance, *E. radiata* in Tasmania tends to exhibit larger thalli and more developed reproductive structures compared to those in New South Wales, likely due to lower sea surface temperatures [59]. In addition, light intensity is a critical determinant of its growth and reproductive success, with prolonged reductions in light availability leading to markedly diminished performance [60]. In Western Australia, specimens from Kalbarri were on average 65.5 cm longer than those from Sydney, reflecting geographic influence on morphology and suggesting phenotypic plasticity [57,61]. However, the polysaccharide composition showed no consistent pattern across regions, with observed variations likely attributable to site-specific environmental conditions [62]. Light is also a crucial factor regulating the growth and reproductive performance of *E. radiata*. While most studies report a negative correlation between lamina surface area and area-to-volume ratio with increasing light intensity—interpreted as an adaptive response—*E. radiata* displayed the opposite trend [63]. It has been suggested that under prolonged low-light conditions, much of the photosynthetically derived energy is allocated to thallus development rather than stipe elongation.

As one of the most widely distributed and abundant kelp species and a key habitat-forming macroalga in Australasia, *E. radiata* nonetheless faces significant threats and is projected to experience further declines. However, recent studies suggest that thermal adaptation in its gametophytes may confer some resilience to future ocean warming [64].

### 2.4. Hormosira banksii

*Hormosira banksii*, commonly known as Turner, is a large, dioecious, perennial and fucoid brown alga commonly found from King George Sound on Australia’s temperate western coast to areas near New South Wales, with high abundance around Tasmania [29]. It forms a dense monotypic canopy in the mid to low intertidal zone, characterized by fluid-filled vesicles that modulate microclimate conditions and support local intertidal ecology [65].

The species is inhibited only on the lower shore, and a considerable proportion of the vertical range of the intertidal rock [66]. It is capable of year-round reproduction, with peak reproductive activity in warmer seasons. Although its temperature tolerance is high—surviving conditions up to 40 °C and as low as 9 °C—its growth rate is notably slow [66,67,68,69]. Morphological variability is often pronounced and mainly associated with vesicle morphology [68]. Despite its environmental tolerance, *H. banksii* is highly susceptible to anthropogenic and natural disturbances such as storm events [70], pollution [71], and trampling [72]. These factors significantly impact its survival due to its inherently slow growth and limited capacity for rapid recovery [65,73]. As mentioned, *H. banksii* is sensitive to pollution. Research has investigated whether another algal species, *Corallina officinalis*, could limit the dominance of *H. banksii* following improvements in water quality. The findings indicated interspecific competition between the two species, with *H. banksii* dominating rocky substrates, suggesting that its presence is constrained in such habitats. Other studies, however, reported that *H. banksii* accumulates primarily as beach wrack on sandy shores rather than rocky ones, with greater abundance in summer compared to autumn [74]. Genetically, *H. banksii* is divided into three groups—western (Peronia), central (Maugea), and eastern (Flindersia). The Bass Strait, located between mainland Australia and Tasmania, represents the transitional zone among these groups. Despite its importance, this region has often been overlooked in genetic studies of the species. A focused study revealed that haplotypes typical of the western and eastern groups are largely absent in the Bass Strait, suggesting that *H. banksii* populations there originated from nearby refugia [75]. These findings align with patterns observed in other marine species, underscoring the role of biogeographic barriers in shaping the genetic structure of coastal organisms.

### 2.5. Phyllospora comosa

*Phyllospora comosa* is a large, fast-growing, perennial brown alga that forms dense canopy structures, offering shelter and food for numerous marine organisms [29,76]. The forests it forms support ecologically and economically important species such as abalone and spiny lobster, making *P. comosa* a valuable species for both environmental conservation and commercial exploitation [28,77]. However, *P. comosa* is highly sensitive to water quality. It vanished from nearshore regions of Sydney during the 1980s, likely due to extensive urban wastewater discharge at the time [76]. Among brown algae, its seedlings are particularly vulnerable to poor water quality [78]. In addition to environmental sensitivity during reproduction, it is prone to fungal infections, which can cause stem rot and necrosis, resulting in significant population declines [79]. As a candidate for commercial aquaculture, optimizing cultivation and improving its survival rate under artificial conditions is crucial. Cumming et al. [80] found that the germination rate of *P. comosa* varied significantly (35–90%) under different controlled conditions, while embryonic mortality after 7 days exceeded 65%, highlighting the need for further studies to enhance artificial propagation techniques. The physiological responses of *P. comosa* to marine heatwaves were examined under two ocean scenarios (current and projected future conditions) [81]. Results showed that *P. comosa* exhibited reduced net photosynthetic rates and increased fatty acid saturation under both conditions. Under present-day conditions, respiration rates increased, whereas under future conditions, the carbon dioxide–concentrating mechanism was reduced. In laboratory experiments, the adverse effects of heatwaves were reversed within seven days; however, the long-term ecological consequences in natural environments require further investigation.

**Table 1:** Characteristics, Distribution, Reproductive Traits, and Environmental Requirements of Five Native Brown Algae Species.

Species	Characteristics	Distribution in Australia	Reproduction	Environmental Requirements
*Cystophora torulosa*	Sausage-shaped leaves, single disc-shaped fixative, long and serrated stipe, thick and branched lateral branches, tightly clustered. The color is brownish brown [29]	From Apollo Bay to Wilsons Promontory in Victoria, around the Bass Straight Islands and Tasmania [82]	Monoecious [36]	Adhere to low tide zones up to 30 m deep reefs, temperate and cold temperate zones [82], prefer moderate wave conditions.
*Durvillaea potatorum*	Dark brown leaf-like body, medium brown tender buds, 2–10 m long (usually 2–8 m), with cone-shaped to wide cone-shaped fixatives with a massive discoid to broadly conical holdfast. 5–50 cm long and 2–12 cm in diameter terete stipe. No internal honeycomb structure [38].	Around King island [83] and Tasmania [84]. The west of Wilson Promontory [38].	Dioecious [85]	Located in an environment of low intertidal and shallow subtidal, rocky substrate, and ocean waves [39]. High turbulence is fluent in reproduction [40]. It often grows in cold temperate waters and is susceptible to the influence of ice scour before disappearing [41].
*Ecklonia radiata*	The stipe is straight and flat, with lateral branches located on both sides, wide and flat. The leaves are brownish wavy in color [29].	Can be found on subtidal rocky substrate from ∼27° S to 48° S [56]. From Houtman Abrolhos on the west coast of Australia, along the south coast to near Queensland on the east coast, including Tasmania, Moreton Island [55].	Dioecious [56]	Located in the low intertidal to a depth of 40 m, it is distributed in temperate and subtropical regions and often lives in water at 8–25 °C [56].
*Hormosira banksii*	Composed of vesicles containing air and liquid [86], they are irregularly spotted circular or elliptical in shape, and can grow up to 30 cm in length. The disc-shaped holder has simple branches. Color brown or dark brown [29].	Can be found from King George’s Sound on the west coast of Australia, along the southern coastline to Coffs Harbor in New South Wales, and also around Tasmania [87].	Dioecious [67]	Commonly found in higher intertidal zones and along the middle and lower coasts [86], it is more resistant to dryness, prefers shelter from wind and moderate wave conditions, and dislikes exposed environments [88].
*Phyllospora comosa*	The main branch is flat, with many closely arranged lateral branches, smooth lateral leaves, and toothed edges. There are vesicles on the short branches, which are randomly distributed with the lateral leaves. Disk-shaped elliptical fixator. Dark brown [29,89].	Along the coast of Victoria to the coast of New South Wales, including Tasmania [89]	Dioecious [29]	Usually inhabiting shallow subtidal reefs at 0–10 m in temperate zones [89]. High requirements for water quality and high sensitivity to sewage [78].

## 3. Nutritive Value of Five Australian Native Brown Algae

### 3.1. Protein

With ongoing economic development and population growth, global demand for protein is steadily increasing. Conventional protein sources, such as meat and dairy products, are associated with environmental concerns and resource limitations [90]. Therefore, identifying new, sustainable, and nutrient-dense protein sources is of increasing importance to support global dietary needs [91]. Seaweed, which is widely available, environmentally friendly, and rich in nutritional components including proteins, has attracted considerable attention as a promising alternative to traditional protein sources [92]. As a plant-based protein source, seaweed provides essential amino acids, making it particularly valuable for vegetarian and vegan populations [93]. The protein content in seaweed is influenced by species, seasonality, and growth conditions. In general, brown algae contain less than 15% protein on a dry weight basis, which is lower than that found in red and green algae [94]. Nonetheless, proteins and amino acids derived from seaweeds exhibit a wide range of pharmacological activities, such as antioxidant and anti-inflammatory effects [95], lipid regulation, and blood pressure reduction. These attributes may contribute to immune enhancement and cardiovascular health [96]. Seaweed protein is characterized by high digestibility and bioavailability, making it easier for the human body to absorb and metabolize [97]. Additionally, its low caloric content makes it suitable for individuals with digestive sensitivities or those aiming for weight management [98].

A comparative study was conducted on five native Australian brown algae species during autumn and winter [28], and the results are summarized in Table 2. Overall, protein content was found to be higher in winter than in autumn, aligning with findings from other studies [99]. An exception was observed in *Hormosira banksii*, which exhibited the highest protein concentration (87.90 mg/g dry weight) in autumn samples, contrary to the seasonal trend. This anomaly may be attributed to interspecific differences or variable environmental conditions, such as temperature and nutrient availability [66,100]. Ash and crude fiber contents showed no significant seasonal variation in most species, with the exception of *H. banksii*, whose ash content in autumn was approximately five times greater than in winter. In contrast, the nitrogen-free extract (NFE) content was consistently higher in winter across all species. The brown algae species analyzed demonstrated considerable nutritional value: moderate to high protein content provides essential amino acids [91]; high ash content indicates mineral richness, supplying vital trace elements [101]; and the presence of crude fiber and NFE supports intestinal motility, offering dietary fiber and carbohydrates that are beneficial for gut health [102]. These nutritional attributes highlight the potential of these brown algae as valuable food and nutraceutical resources. Table 2 summarizes this comprehensive research and compared with other edible seaweeds for proximate composition. Overall, protein content was found to be higher in winter than in autumn, aligning with findings from other studies [99]. An exception was observed in *Hormosira banksii*, which exhibited the highest protein concentration (87.90 mg/g dry weight) in autumn samples, contrary to the seasonal trend. This anomaly may be attributed to interspecific differences or variable environmental conditions, such as temperature and nutrient availability [66,100]. Ash and crude fiber contents showed no significant seasonal variation in most species, with the exception of *H. banksii*, whose ash content in autumn was approximately five times greater than in winter. In contrast, the nitrogen-free extract (NFE) content was consistently higher in winter across all species. The brown algae species analyzed demonstrated considerable nutritional value: moderate to high protein content provides essential amino acids [91]; high ash content indicates mineral richness, supplying vital trace elements [101]; and the presence of crude fiber and NFE supports intestinal motility, offering dietary fiber and carbohydrates that are beneficial for gut health [102]. These nutritional attributes highlight the potential of these brown algae as valuable food and nutraceutical resources.

### 3.2. Lipids

Lipids are key components of biological membranes and serve as essential nutrients, providing energy, fat-soluble vitamins, and functional support for growth, cellular integrity, and physiological health [108,109,110,111]. Fatty acids, the main constituents of lipids, can be categorized into saturated fatty acids (SFA), monounsaturated fatty acids (MUFA), and polyunsaturated fatty acids (PUFA) [112]. Among them, polyunsaturated fatty acids—specifically Omega-3 and Omega-6—are considered essential because they cannot be synthesized by the human body and must be obtained from dietary sources [113]. Omega-3 fatty acids are primarily found in fish and certain plant sources, while Omega-6 fatty acids are abundant in vegetable oils [112,114]. Algae are among the most important natural producers of PUFA [115]. Although their total lipid content is relatively low compared to marine animals such as fish [116], algae contribute significantly to the marine food web, serving as the foundational PUFA source for fish. Consequently, the polyunsaturated fatty acids found in fish oil are, to a large extent, derived metabolically from algae [117]. Currently, fish oil is the primary source of dietary Omega-3 for humans; however, the sustainability of fisheries is under increasing pressure due to overfishing, environmental stress, and climate-related disruptions [118]. For example, adult salmon returns have declined markedly in recent years [119], and Western Baltic cod populations have reached a threshold of unsustainable exploitation [120]. As a result, there is an urgent need to identify alternative, stable sources of PUFA. Seaweeds, particularly brown macroalgae, represent a promising, renewable, and environmentally sustainable option.

The total lipid content in seaweeds generally does not exceed 5% of dry weight and varies by species, season, and environmental conditions [121,122,123]. Lipid profiles of the five selected native brown algae were compiled from studies conducted in Tasmania, Victoria, and New South Wales [28,124,125,126], and are summarized in Table 3. Total fatty acid (TFA) content averaged around 1% of dry weight across all five species. No significant differences were observed among species in terms of SFA or MUFA content, whereas PUFA accounted for approximately 50% of total fatty acids in most species. However, regional differences were observed within the same species. For instance, *Ecklonia radiata* and *Hormosira banksii* from New South Wales exhibited nearly double the SFA content compared to samples from Tasmania and Victoria. Conversely, PUFA levels were notably higher in Tasmanian and Victorian specimens, particularly in *E. radiata*. *Cystophora torulosa* had the lowest MUFA content, followed by *Durvillaea potatorum*, while the other three species exhibited approximately 20% MUFA. PUFA levels were similar in *C. torulosa*, *D. potatorum*, *E. radiata*, and *H. banksii*, with *C. torulosa* exhibiting the highest proportion. Importantly, the n-6/n-3 fatty acid ratio for all five species was below 3.8, and in most Tasmanian and Victorian samples, especially those of *C. torulosa*, *D. potatorum*, *E. radiata*, and *H. banksii*, the ratio approached 1. This is noteworthy because the recommended dietary ratio of n-6 to n-3 ranges from 2.5:1 to 4:1. In contrast, modern diets often result in n-6/n-3 ratios exceeding 10:1, which is associated with increased risk of chronic diseases such as cardiovascular disease, inflammation, obesity, and metabolic disorders [127,128,129,130]. This indicates that native Australian brown algae have the potential to serve as high-quality dietary lipid sources. In Table 3, a comparison of the five native Australian brown seaweeds with the commercial species *Laminaria digitata*, *Sargassum fusiforme*, and *Undaria pinnatifida* shows that their SFA, MUFA, and PUFA contents are broadly comparable. Although the n-6/n-3 ratio of the Australian species is slightly higher, it remains close to 1 across all cases, suggesting that these native brown seaweeds possess strong commercial potential comparable to that of established commercial species.

A study conducted among 1301 adolescents aged 13–15 years in Western Australia reported a mean Omega-3 index of 4.90 ± 1.04%, with 15.6% classified as high risk for degenerative diseases [131]. Another survey of 251 adolescents aged 15–17 years from regional New South Wales found that 53% had Omega-3 indices below the optimal threshold (>8%), and 3% were below 4% [132]. These findings highlight suboptimal Omega-3 intake among Australian adolescents, which has significant implications for brain development and neurological health [133,134]. Incorporating seaweed into daily diets may therefore be a viable strategy to enhance Omega-3 intake and support adolescent health.

It is important to note that lipid composition in seaweeds is influenced by seasonal, geographic, and post-harvest conditions. Significant differences were observed between freshly harvested *D. potatorum* samples, sun-dried beach samples, and commercially processed materials [126]. Therefore, standardized harvesting, processing, and cultivation protocols are essential to ensure consistent lipid quality in future seaweed-based products.

**Table 3:** Fatty Acid Profiles of Eight Brown Seaweed Species.

Species		TFA	SFA	MUFA	PUFA	n-6/n-3	EPA + DHA	ALA	Location	References
*Cystophora torulosa*		0.8 ± 0.1 % dry weight	30.2 ± 0.7	17.8 ± 0.6	51.6 ± 1.2	1.4 ± 0.2	3.6 ± 0.6 mg/10 g DW	5.8 ± 0.8 mg/10 g DW	Tasmania	[124]
		13.98 ± 2.13 mg/g DW	26.04 ± 0.32	12.94 ± 0.19	59.45 ± 0.19	0.72 ± 0.01	6.56 ± 0.16	8.07 ± 0.06	Victoria	[28]
*Durvillaea potatorum*		1.87 ± 0.48 mg/g DW	33.55 ± 1.51	15.46 ± 1.48	49.70 ± 1.14	0.94 ± 0.06	6.38 ± 0.19	11.21 ± 1.14	Victoria	[28]
	Fresh	13 mg/g DW	12.1	11.6	56.9	0.73	5.1	23.8	Tasmania	[126]
	Factory-Dried	5 mg/g DW	28.2	21	37.3	1.25	3.7	10.2	Tasmania	[126]
	Beach	1 mg/g DW	39.3	18.3	22.2	2.13	1.9	4.3	Tasmania	[126]
*Ecklonia radiata*		1.2 ± 0.2 % dry weight	25.0 ± 1.9	22.3 ± 2.1	52.3 ± 4.0	1.1 ± 0.2	6.8 ± 1.5 mg/10 g DW	7.9 ± 1.4 mg/10 g DW	Tasmania	[124]
			50.7	33	16.3	3	2.3	1.8	New South Wales	[125]
		7.47 ± 0.34 mg/g DW	32.29 ± 1.00	20.95 ± 0.31	45.50 ± 1.12	0.98 ± 0.06	6.43 ± 0.27	5.69 ± 0.44	Victoria	[28]
*Hormosira banksii*		1.0 ± 0.1 % dry weight	31.3 ± 0.9	21.1 ± 1.4	47.4 ± 0.7	1.0 ± 0.2	9.2 ± 1.2 mg/10 g DW	8.9 ± 3.0 mg/10 g DW	Tasmania	[124]
			40.6	24.6	34.8	1.5	5.8	7.4	New South Wales	[125]
		3.93 ± 0.26 mg/g DW	31.48 ± 0.42	19.06 ± 0.27	47.81 ± 0.26	1.05 ± 0.09	6.38 ± 0.32	8.98 ± 0.69	Victoria	[28]
*Phyllospora comosa*			42	20.8	37.2	3.8	3.9	3.9	New South Wales	[125]
		2.53 ± 0.19 mg/g DW	36.63 ± 0.65	20.29 ± 0.46	40.81 ± 0.97	1.83 ± 0.02	5.14 ± 0.16	5.11 ± 0.30	Victoria	[28]
*Laminaria digitata*		0.11 ± 0.01 –1.03 ± 0.08 %DW	28.40 ± 1.22 –47.57 ± 4.50	14.31 ± 5.05 –17.63 ± 0.18	32.93 ± 4.22 –49.93 ± 3.06	0.65 ± 0.03 –0.98 ± 0.03	-	-	Ireland	[135]
*Sargassum fusiforme*		-	36.78	18.09	45.13	1.2	-	-	China	[105]
*Undaria pinnatifida*		-	4.47 ± 0.64	2.22 ± 0.12	11.2 ± 1.06	0.71	-	-	New Zealand	[136]

Note: Unless otherwise specified, all units are % of total fatty acid. TFA: Total Fatty Acids; SFA: Saturated Fatty Acids; MUFA: Monounsaturated Fatty Acids; PUFA: Polyunsaturated Fatty Acids; EPA: Eicosapentaenoic Acid; ALA: Alpha-Linolenic Acid; DW: dry weight.

### 3.3. Trace Elements

Seaweeds are rich sources of essential dietary minerals, often containing concentrations at least ten times higher than those found in terrestrial plants, making them important contributors to daily micronutrient intake [137]. Among these, iodine, iron, zinc, magnesium, calcium, and selenium are the most frequently discussed due to their physiological importance and the adverse health effects associated with their deficiencies. For instance, iodine deficiency can lead to thyroid dysfunction and impaired physical development [138,139]; zinc deficiency is linked to stunted growth and weakened immune response [140]; and iron deficiency is a leading cause of anemia [141,142].

In Australia, zinc deficiency is the most prevalent micronutrient insufficiency, largely due to the limitations of land-based agriculture [143]. Additionally, iron deficiency is more pronounced among women, and calcium intake remains below recommended levels across all age groups, with inadequacy increasing with age [144]. Thus, ensuring a balanced intake of dietary minerals is critical for public health. Including seaweeds in the diet provides a sustainable and effective means of supplementing essential micronutrients.

A mineral composition analysis of the five selected native Australian brown algae revealed varying concentrations of 12 trace elements [145], with selected data presented in Table 4. *Cystophora torulosa* exhibited the highest concentrations of calcium (7461.4 ± 795.8 mg/kg), chromium (6.3 ± 2.6 mg/kg), and iron (334.1 ± 115.2 mg/kg). *Durvillaea potatorum* was found to have elevated levels of magnesium, sodium, and zinc. In contrast, *Ecklonia radiata* had the lowest concentrations of several elements, including chromium, iodine, iron, manganese, and sodium. *Hormosira banksii* displayed the highest concentrations of potassium (38,994.1 ± 4967.4 mg/kg), magnesium (11,476.5 ± 1461.1 mg/kg), manganese (49.8 ± 29.3 mg/kg), selenium (1.0 ± 0.1 mg/kg), and sodium (55,276.1 ± 14,501.1 mg/kg). Meanwhile, *Phyllospora comosa* recorded the highest levels of iodine (887.8 ± 100.0 mg/kg) and zinc (33.6 ± 9.5 mg/kg). These results highlight substantial interspecies variability in mineral content. For example, *C. torulosa* can be considered a valuable dietary source of calcium, *H. banksii* is rich in magnesium, and *P. comosa* offers a high level of zinc. Therefore, dietary formulations incorporating a mixture of these brown algae could provide a more comprehensive mineral profile and optimize nutritional benefits.

However, special attention must be paid to the iodine content in seaweeds. While iodine is essential, excessive intake may lead to thyroid dysfunction [146]. In Australia, iodine is already added to staple foods such as dairy products and bread, ensuring a baseline dietary intake for the general population [147]. Consequently, additional iodine from seaweed must be carefully managed. The Australian Government Department of Agriculture, Fisheries and Forestry has classified brown algae as a high-risk food due to potential excess iodine levels. Imported brown algae must undergo iodine testing and comply with the maximum allowable limit of 1000 mg/kg dry weight [148]. In addition, inorganic arsenic restrictions apply exclusively to *Sargassum fusiforme*, for which the maximum permissible concentration of inorganic arsenic is set at 1 mg/kg, based on an assumed hydration level of 85% [148]. In industrial processing, hydrothermal treatment has been shown to significantly reduce iodine and arsenic concentrations in *Phyllospora comosa*, bringing levels into a safe range. However, this method can lead to a biomass loss of up to 50% [149].Therefore, optimization of processing techniques remains essential to preserve both safety and nutritional value in seaweed-based food products.

### 3.4. Polysaccharides

Seaweeds contain substantial amounts of polysaccharides that serve essential roles in macroalgal cells, including structural support and energy storage [150]. The polysaccharide content of macroalgae ranges from 4% to 76% of dry weight, depending on species and environmental factors [25,151]. Different algal groups are characterized by distinct types of polysaccharides. Red algae are known for their production of carrageenan, agar, xylan, and porphyran, which are widely used in food, agriculture, cosmetics, and pharmaceuticals [152]. Green algae primarily produce ulvans, a class of sulfated polysaccharides that have applications in fertilizers, biofuels, and bioremediation. Brown algae mainly contain laminarin, alginates, and fucoidan, which have been extensively utilized in food, fertilizer, and pharmaceutical industries [152].

Laminarin serves as a storage compound and exhibits diverse bioactivities, such as anti-inflammatory effects and pro-apoptotic activity against human colon cancer cells [153]. Fucoidan is a sulfated polysaccharide primarily composed of α-*L*-fucose, accounting for approximately 2–20% of the dry weight of brown algae, depending on species, season, and extraction method. It exhibits a range of biological activities, including antioxidant properties, modulation of gut microbiota, and potential therapeutic effects in models of Alzheimer’s disease [154]. Alginates, linear anionic polysaccharides composed of β-*D*-mannuronic acid (M) and α-*L*-guluronic acid (G) linked via 1,4-glycosidic bonds, can constitute up to 40% of the dry weight of brown algae and are commonly processed into soluble sodium alginate for commercial use [155].

The fucoidan content of *Hormosira banksii* ranges from 2.1% to 7.7% of dry biomass, comparable to other commercial brown algae species. However, it may not be suitable for co-cultivation with oysters, and further research is required to develop compatible aquaculture protocols [156]. Using consistent extraction methods, comparative studies of *Durvillaea potatorum* and *Ecklonia radiata* showed that the sequence of fucoidan and alginate extraction had minimal impact on final yields. Notably, the fucoidan content of *E. radiata* reached 29.7% in winter and 18.2% in autumn, comparable to established commercial species such as *Macrocystis pyrifera* and *Undaria pinnatifida*. Meanwhile, *D. potatorum* consistently yielded around 15% fucoidan [157].

Further characterization of the fucoidan and laminarin extracted from *Phyllospora comosa*, *D. potatorum*, and *E. radiata* demonstrated that these compounds were structurally degraded under simulated gastrointestinal conditions. Despite this, both fucoidan and laminarin retained significant antioxidant and antidiabetic activities, although their biological activity may be reduced post-digestion [158].

In addition, employing an acid pre-treatment prior to conventional alkaline extraction enabled the isolation of both acid-extractable alginate (acid-A1) and alkaline-extractable alginate (alkaline-A2) from *D. potatorum*, resulting in a 4.6% increase in total polysaccharide yield compared to traditional methods [159]. Polysaccharides, polyphenols, and peptides extracted from *E. radiata* and *P. comosa* collected in New South Wales have demonstrated inhibitory activity against enzymes associated with metabolic syndromes, including angiotensin-I-converting enzyme, α-amylase, and lipase, suggesting potential roles in the management of hypertension, diabetes, and obesity [160].

Nutritional profiling of different anatomical parts of *P. comosa* revealed exceptionally high carbohydrate content (64–68%), particularly in the stipes, slightly surpassing that of *D. potatorum* (64%) and representing one of the highest fucoidan concentrations reported among temperate brown algae [161]. These findings highlight the industrial potential of these species for alginate and fucoidan production, with *P. comosa* showing particularly strong promise, although further optimization of cultivation and extraction methods is needed.

Collectively, these studies demonstrate that native Australian brown algae possess diverse bioactive polysaccharides with antioxidant, anti-inflammatory, antidiabetic, and antihypertensive properties. Moreover, their polysaccharide yields are comparable to or even exceed those of established commercial species, underscoring their biomedical relevance and potential high commercial value.

### 3.5. Other Bioactive Components and Applications

In addition to the aforementioned nutrients, brown algae are rich in a wide array of other bioactive compounds, including phlorotannins, bromophenols, terpenes, and sterols, which exhibit various biological activities such as antioxidant, antibacterial, anticancer, and antidiabetic effects [162,163,164,165,166]. These compounds have potential applications in functional foods, pharmaceuticals, cosmetics, and other industrial sectors [164,165]. Phenolic extracts from *Durvillaea potatorum*, *Phyllospora comosa*, and *Ecklonia radiata* have demonstrated significant inhibitory effects on protein denaturation, suggesting anti-inflammatory, antidiabetic, and antiproliferative potential [167]. Furthermore, *E. radiata* and *P. comosa* have shown significantly higher nitric oxide (NO) inhibitory activity compared to other native brown algae, indicating promising anti-inflammatory capacities [125]. Although some phytochemical studies have been conducted on Australian macroalgae [163], in-depth research focusing on the selected edible brown algae species remains limited. Comprehensive investigations into their bioactive compound profiles, pharmacological potential, and food-grade applications are still needed to realize their full value. These compounds have potential applications in functional foods, pharmaceuticals, cosmetics, and other industrial sectors [164,165]. Phenolic extracts from *Durvillaea potatorum*, *Phyllospora comosa*, and *Ecklonia radiata* have demonstrated significant inhibitory effects on protein denaturation, suggesting anti-inflammatory, antidiabetic, and antiproliferative potential [167]. Furthermore, *E. radiata* and *P. comosa* have shown significantly higher nitric oxide (NO) inhibitory activity compared to other native brown algae, indicating promising anti-inflammatory capacities [125]. Although some phytochemical studies have been conducted on Australian macroalgae [163]. In-depth research focusing on the selected edible brown algae species remains limited. Comprehensive investigations into their bioactive compound profiles, pharmacological potential, and food-grade applications are still needed to realize their full value.

Among the five native species, *Durvillaea potatorum* stands out as the most widely studied and commercially utilized brown alga in Australia. It has been explored for agricultural applications since the 1970s [168]. Recent studies highlight the role of *D. potatorum* extracts in enhancing crop nutrition and growth performance. For instance, when applied to broccoli, the extract significantly increases seedling leaf number and biomass accumulation [169]. It also exhibits protective effects against root and fungal infections [170]. In viticulture, foliar application of *D. potatorum* extracts has been shown to improve grape yields by an average of 14.7%, resulting in enhanced economic returns [171]. Similar effects have been observed in sugarcane cultivation, with yield increases of 17%, sugar content improvements, and overall economic gains of up to 18% [172]. In avocado production, treatment with this extract improved fruit quality and yield, with an average per-tree yield increase of 38% and fruit firmness enhancement by 22%, leading to a 24% overall increase in revenue [173]. In strawberry cultivation, *D. potatorum* extract promoted root development, increasing secondary root density by up to 22%, marketable runner yield by 8–19%, and fruit yield by 8% [49]. These findings collectively suggest that *Durvillaea potatorum* possesses outstanding agronomic and economic potential. Currently, it is the most extensively researched and industrially applied brown algae species in Australia.

## 4. Australians’ Attitudes Towards Seaweed and Seaweed Products

Birch et al. [174] administered a seaweed consumption questionnaire to 521 Australians across various demographics, including gender, age, education, income, and ethnicity. The results showed that nearly three-quarters of respondents had previously consumed or tried seaweed; however, overall consumption frequency remained low, with only 37% reporting consumption at least once per month over the past 12 months. Gender did not significantly influence seaweed intake frequency, whereas education, income, and age were more impactful. Higher levels of education and income were associated with increased likelihood and frequency of seaweed consumption, while age exhibited a negative correlation. The most commonly consumed seaweed product was sushi (70.6%), followed by crackers, soups, and snacks. Key barriers to consumption included a lack of knowledge, limited availability, and cost.

In a larger survey, Young et al. [175] assessed attitudes among 1403 Australians under the age of 30. The findings revealed that young consumers appreciated the flavor, nutritional value, and health benefits of seaweed products but were deterred by limited accessibility, high prices, and food neophobia. Seaweed was generally preferred in snack form. A common challenge across age groups was the lack of familiarity with seaweed-derived products and their nutritional attributes. To promote seaweed consumption in Australia, increased public awareness campaigns, educational outreach, and product sampling are recommended. Encouraging local seaweed cultivation and reducing reliance on imports can also help stabilize pricing and enhance domestic market sustainability [24]. Recognizing the potential of a homegrown seaweed industry, the Australian government has invested AUD 8.1 million through the Fisheries Research and Development Corporation (FRDC) to support its development [176].

Seaweed can be incorporated into various food products, including meat items (e.g., steak, sausages, fish-based products), where it improves shelf life, nutritional quality, and flavor. It is also used in bread and fermented dairy products [116,177,178]. Among these applications, consumer preference tends to favor dried seaweed incorporated into bread or used as a standalone product, while seaweed additions to yogurt and sausages are less well-received [179]. Seaweed is especially important for vegetarians, whose primary protein sources—grains and legumes—often fall short of fulfilling daily amino acid requirements. Brown seaweeds, with their relatively high protein content and complete amino acid profiles, represent a valuable protein alternative. However, appropriate monitoring of daily iodine intake is essential to avoid excessive iodine consumption and related thyroid complications [91,180,181,182].

A sensory evaluation study compared five selected native Australian brown algae with the commercially available seaweed *Sargassum fusiforme* [28]. Dishes such as soups made from *Durvillaea potatorum*, *Ecklonia radiata*, and *Phyllospora comosa* showed no significant differences in consumer preference compared to *S. fusiforme*. However, salads prepared with *Cystophora torulosa* received significantly lower ratings than those made with *S. fusiforme*, which achieved the highest consumer preference scores. These results suggest that Australian consumers show comparable interest in foods prepared with native wild seaweeds and with commonly available commercial species. Therefore, the selected native brown algae possess considerable commercial potential. Nevertheless, further studies are required to establish industry standards, optimize marketing strategies, and promote responsible consumer practices to fully realize the economic potential of Australia’s native seaweeds.

According to the Australian Seaweed Industry Blueprint, the Australian seaweed market is developing domestically and internationally, covering different product areas such as food and human nutrition products, animal feed supplementation, cosmetics, biofertilizer, bioplastics and bioremediation [183]. Among Australia’s brown seaweed species, *Ecklonia radiata*, known as Golden Kelp, has been used for the production of various commercial products such as snacks, superfoods, food garnishes and skincare products (https://www.seahealthproducts.com.au/, accessed on 19 September 2025). Besides the brown seaweed species mentioned in this article, Australia-based *Undaria pinnatifida* has also been commercialized as a food seasoning product (https://www.phycohealth.com/, accessed on 19 September 2025). Other than human application, Australian-grown *Ascophllum nodosum* and *Durvillaea potatorum* have also been produced as animal feed supplements (https://www.seaperia.com/, https://auskelp.com.au/, accessed on 19 September 2025).

## 5. Conclusions

Australia possesses abundant marine resources, including a diverse array of unique seaweed species that contribute significantly to its complex coastal ecosystems. This review focused on five native brown algae species—*Cystophora torulosa*, *Durvillaea potatorum*, *Ecklonia radiata*, *Hormosira banksii*, and *Phyllospora comosa*—and comprehensively evaluated their biological characteristics, nutritional composition, potential applications, and market prospects. Environmental factors such as temperature, water quality, and light availability were found to significantly influence the growth and distribution of these algae. Among them, *Ecklonia radiata* and *Hormosira banksii* demonstrated strong temperature tolerance; however, *Hormosira banksii* exhibited slower growth rates, while *Phyllospora comosa* grew more rapidly but was more sensitive to water quality fluctuations. Nutritional analyses indicated that *Durvillaea potatorum* is particularly rich in protein, lipids, trace elements, and polysaccharides, and it has already demonstrated high commercial value, especially in agricultural applications. In addition, *Ecklonia radiata* and *Phyllospora comosa* also exhibited significant bioactive potential and industrial value, comparable to commercial seaweed species. Consumer studies suggest that Australians exhibit relatively high acceptance of seaweed and seaweed-based products, particularly when incorporated into familiar food formats. However, general awareness of the nutritional and functional benefits of seaweed remains limited, highlighting the need for targeted education and marketing strategies to support broader market adoption.

To foster the sustainable development of the Australian seaweed industry, further research and innovation are needed to optimize cultivation techniques, diversify product applications, and enhance public understanding. Among the species assessed, *Durvillaea potatorum* has already undergone partial commercialization and serves as a model for future development. With appropriate support, *Ecklonia radiata* and *Phyllospora comosa* also hold strong potential for industrial exploitation, offering new opportunities for nutrition, health, and sustainable resource use in Australia.

## Figures and Tables

**Table 2:** Protein, Ash, Crude Fibre, and NFE Levels in Ten Algae Species During Autumn and Winter.

Species	Seasons	*Cystophora torulosa*	*Durvillaea potatorum*	*Ecklonia radiata*	*Hormosira banksii*	*Phyllospora comosa*	*Palmaria palmata*	*Laminaria digitata*	*Sargassum fusiforme*	*Undaria pinnatifida*	*Porphyra purpurea*
Classification		Brown	Brown	Brown	Brown	Brown	Red	Brown	Brown	Brown	Red
Protein mg/g dry tissue	Autumn	54.69 ± 1.70	30.78 ± 3.39	63.69 ± 0.11	87.90 ± 1.47	57.02 ± 2.37	8.40 ± 0.05–16.88 ± 0.11% dw	6.9 ± 1.1% dw	3.90%	16.76 ± 0.06–28.19 ± 0.03% dw	33–47% dw
	Winter	60.75 ± 1.10	50.53 ± 5.73	84.67 ± 6.50	30.78 ± 1.44	56.06 ± 1.80					
Ash mg/g	Autumn	131.35 ± 2.78	160.58 ± 9.99	128.52 ± 6.66	156.02 ± 0.30	133.19 ± 2.69	11.14 ± 0.10–39.97 ± 0.23% dw	31.6 ± 7.1% dw	31.23%	17.23 ± 0.12–43.92 ± 0.02% dw	21.3% dw
	Winter	134.92 ± 1.73	168.61 ± 5.64	121.01 ± 5.64	195.76 ± 8.04	129.70 ± 3.09					
Crude fibre mg/g	Autumn	132.65 ± 37.86	43.19 ± 6.40	127.30 ± 3.95	220.07 ± 90.92	86.14 ± 2.57	8.09 ± 0.02–12.79 ± 0.04% dw	-	-	4.68 ± 1.17 –6.14 ± 0.91% dw	7.5% dw
	Winter	104.95 ± 13.81	30.45 ± 3.59	158.07 ± 17.23	43.98 ± 5.53	79.04 ± 2.04					
NFE mg/g	Autumn	104.95 ± 13.81	30.45 ± 3.59	158.07 ± 17.23	43.98 ± 5.53	79.04 ± 2.04	-	-	-	-	-
	Winter	468.85 ± 41.45	637.32 ± 20.82	571.64 ± 11.52	439.70 ± 89.54	595.83 ± 6.50					
ref		[28]	[28]	[28]	[28]	[28]	[103]	[104]	[105]	[106]	[107]

Note: Seaweed production area: Pea Soup, Port Fairy, Victoria, Australia; NFE: Nitrogen-Free Extract.

**Table 4:** Concentrations of Major Minerals in Five Brown Algae Species.

Element Concentration (mg kg^−1^)	*Cystophora torulosa*	*Durvillaea potatorum*	*Ecklonia radiata*	*Hormosira banksii*	*Phyllospora comosa*
Ca	7461.4 ± 795.8	3593.4 ± 73.4	6042.3 ± 408.6	5931.3 ± 469.1	5564.0 ± 144.4
I	58.9 ± 5.9	109.9 ± 41.1	179.0 ± 29.7	221.2 ± 107.2	887.8 ± 100.0
Fe	334.1 ± 115.2	23.8 ± 7.7	67.3 ± 11.2	97.4 ± 56.1	28.4 ± 4.3
Mg	5718.9 ± 321.2	9068.4 ± 307.3	6980.3 ± 236.3	11,476.5 ± 1461.1	8488.5 ± 155.9
Zn	4.9 ± 1.1	16.7 ± 4.1	23.5 ± 10.6	11.0 ± 5.5	33.6 ± 9.5

Note: Data sources [145].

## Data Availability

Data sharing is not applicable.

## Abbreviations

The following abbreviations are used in this manuscript:

NFE	Nitrogen-Free Extract
TFA	Total Fatty Acids
SFA	Saturated Fatty Acids
MUFA	Monounsaturated Fatty Acids
PUFA	Polyunsaturated Fatty Acids
EPA	Eicosapentaenoic Acid
ALA	Alpha-Linolenic Acid
FRDC	Fisheries Research and Development Corporation
DW	Dry weight
